# Eco-engineering effects of *Setaria viridis* and *Panicum bisulcatum Thunb* roots on slope stability under controlled moisture conditions

**DOI:** 10.1038/s41598-025-24096-3

**Published:** 2025-11-18

**Authors:** Jinguo Lyu, Yanli Zhang, Te Dai, Wenqi Wang, Songjiang Sang

**Affiliations:** 1 School of Mechanics and Engineering, Liaoning Technical University, Fuxin, 123000 Liaoning China; 2 College of Environmental Science and Engineering, Liaoning Technical University, Fuxin, 123000 Liaoning China; 3 College of Mining, Liaoning Technical University, Fuxin, 123000 Liaoning China

**Keywords:** Soil reinforcement, Root content, Moisture content, Direct shear test, Ecology, Ecology, Environmental sciences, Plant sciences

## Abstract

The slope protection effect of herbaceous plants is closely related to plant species, root content in the soil, and soil moisture content. This study focuses on the roots of common herbaceous plants, such as *Panicum bisulcatum Thunb* and *Setaria viridis*, on the slopes of the Fuxin Haizhou open-pit coal mine. Field measurements were taken to determine the root content at different soil layer depths, the reinforcement effect of plant roots on soil stability was further examined through theoretical analysis of their mechanical contribution to anti-sliding performance. Laboratory tests were performed to measure the changes in shear strength of root-soil composites under different moisture content conditions. Numerical simulations were used to analyze the slope protection effect of herbaceous plant root systems under varying moisture content conditions. The results showed that the root systems of herbaceous plants increased the soil’s cohesion, restricted lateral deformation, and improved the soil’s shear strength. The cohesion and shear strength of the root-soil composite decreased with increasing moisture content, while the internal friction angle increased slightly, though not significantly. The stability factor of root-soil slopes and bare soil slopes first increased and then decreased when the slope moisture content ranged from 9 to 13%, indicating that herbaceous plants have considerable potential for enhancing shallow slope stability and can be effectively applied in ecological restoration and slope protection in mining areas.

## Introduction

The stability of mine slopes is a common issue in geological hazards, directly affecting the safety and economic benefits of mining operations. It has drawn significant attention from researchers in the relevant fields. In recent years, the reinforcing effect of plant root systems on soil has gained increasing recognition and has gradually been applied in practical engineering projects.

Vegetation restoration and ecological reconstruction are essential measures to mitigate the ecological damage caused by open-pit mining, control the occurrence of shallow-seated slope geological hazards, and achieve sustainable ecological mining^1^. Plant root systems enhance slope stability through both hydrological and mechanical effects. The presence of roots prevents direct rainfall-induced erosion and scouring of slope soil, suppresses surface runoff, and reduces soil loss caused by heavy rainfall and other extreme weather events. Simultaneously, roots provide anchorage and soil reinforcement, restricting lateral deformation of surface soil under stress. For slopes with shallow slip surfaces, well-developed root systems can grow below the slip surface, generating shear resistance between the roots and soil, thereby anchoring the slope soil. When the slip surface is deeper, roots primarily act as reinforcement elements, enhancing the mechanical properties of the soil mass.

Many scholars have conducted extensive research to explore the root reinforcement mechanisms and identify factors influencing root-soil interaction. Researchers worldwide have developed scientific root-soil mechanical models through numerical simulation methods^2,3^ to investigate the mechanical processes of root reinforcement or employed experimental methods to study root distribution patterns^4–7^, growth duration^8,9^, and plant species^10,11^ to examine their effects on soil reinforcement. Wang et al.^12^ found that the soil reinforcement effect of roots is related to root structural types, and classifying root systems can better reveal the underlying reinforcement mechanisms. Sun Qingmin^13^ analyzed the shear strength factors of 16 plant root-soil composites and discovered that various factors strongly correlate with soil cohesion and internal friction angle, with the highest correlation observed between cohesion and dry density. Mao Xurui^14^ conducted direct shear tests on different plant root-soil composites and found that roots mainly enhance soil shear strength by increasing soil cohesion. Zhu Jinqi^15^ quantitatively evaluated the soil reinforcement effect of root systems by analyzing the stress mechanisms of roots at different growth stages based on direct shear tests and root pull-out tests, optimizing the Wu model from a time perspective. Wang Wenqi et al.^16^ regarded root-soil composites as materials capable of improving the shear strength of in-situ soil and proposed a mechanical model for enhanced soil consolidation. Li Xunchang^17^ used FLAC^3D^ software to simulate slopes with Artemisia plants at various angles and calculated slope stability coefficients under different conditions, concluding that plant roots enhance soil mechanical strength. Ekanayake and Phillips^18,19^ introduced an energy-based model for evaluating the soil reinforcement effect of herbaceous plant roots, linking the energy consumed during shear to the shear strength of root-soil composites, inferring root reinforcement effects on soil shear strength from an energy perspective. Although simple to calculate, this model has limited accuracy for root-soil composites with thicker roots.

Despite these advances, important limitations remain in root–soil mechanical modelling. Classical Wu model and their extensions—which conceptualize roots as tensile elements contributing an apparent cohesion—offer a practical engineering route, but they often assume uniform root distributions and may not capture natural spatial heterogeneity. In contrast, fracture-mechanics based approaches such as the Fiber Bundle Model (FBM)^20^ can simulate progressive root failure and better reflect actual failure mechanisms; however, FBM requires extensive datasets (root-diameter and tensile-strength distributions, root counts and spatial arrangement) that are difficult and time-consuming to obtain in the field. Given that the present study focuses on controlled laboratory direct shear tests to systematically observe the relative effects of root content and soil moisture on shear strength, and because the full set of root-system mechanical distribution data required to fully parameterize detailed models (e.g. FBM) was not available, we did not directly input experimental parameters into any numerical model for parameterization. Instead, our analysis is driven by experimental results and uses FBM model concepts of equivalent root cohesion for qualitative and semi-quantitative interpretation, ensuring conclusions are based on observable tests and remain practically applicable.

To facilitate links between our laboratory results and engineering practice, we reference Greenwood’s engineering approach^21^, which represents vegetation effects as an equivalent apparent cohesion together with hydrological terms, enabling routine limit-equilibrium comparisons of factors of safety between vegetated and bare conditions. This provides justification for interpreting experimental findings using an equivalent-root-cohesion concept when detailed mechanistic parameterization is not achievable.

Due to the loose nature of mine spoil, mine slopes are prone to landslides and other geological hazards. Furthermore, the root reinforcement effect on soil is influenced by environmental factors, necessitating further analysis of its stabilizing mechanisms. The primary objectives of this study are to: (1) investigate the reinforcement effects of two common herbaceous plants (Panicum bisulcatum Thunb and Setaria viridis) on slope soil under controlled moisture conditions, (2) quantify the contributions of root content and soil moisture to the shear strength of root-soil composites using indoor direct shear tests and the Mohr–Coulomb criterion, and (3) provide insights for ecological restoration and slope stabilization in mining environments.

This study, based on the Haizhou open-pit coal mine in Fuxin, Liaoning Province, China, selects the most common herbaceous plants on the northern slope—*Panicum bisulcatum Thunb* and *Setaria viridis*—as research subjects. Utilizing root-soil composite indoor direct shear tests combined with the Mohr–Coulomb strength criterion, this study investigates the reinforcement effects of herbaceous plant roots on soil stability. The research findings aim to provide a scientific basis for the ecological restoration of mine slopes.

## Mechanical analysis of root-soil interaction

### Mechanical modeling of root consolidation and soil modification

The soil influenced by root systems can be regarded as a root-soil composite. When the shallow root-soil composite on a slope undergoes critical instability and slides along the slip surface, the Mohr–Coulomb strength criterion, combined with the reinforced soil principle, can be used to analyze the improvement of shear strength in shallow slope soil by herbaceous plant roots. Therefore, the shear strength of the root-soil composite^22^ must satisfy Eq. (1).

1 $$\tau = \sigma \tan \varphi + c + \Delta \tau$$

In the equation, *τ* represents the shear strength of the root-soil composite; *σ* denotes the normal stress of the soil without roots; *φ* is the internal friction angle of the soil without roots; *c* refers to the cohesion of the soil without roots; and ∆*τ* represents the increment of shear strength induced by the root-soil composite, referred to here as the total additional shear strength.

**Fig. 1:**
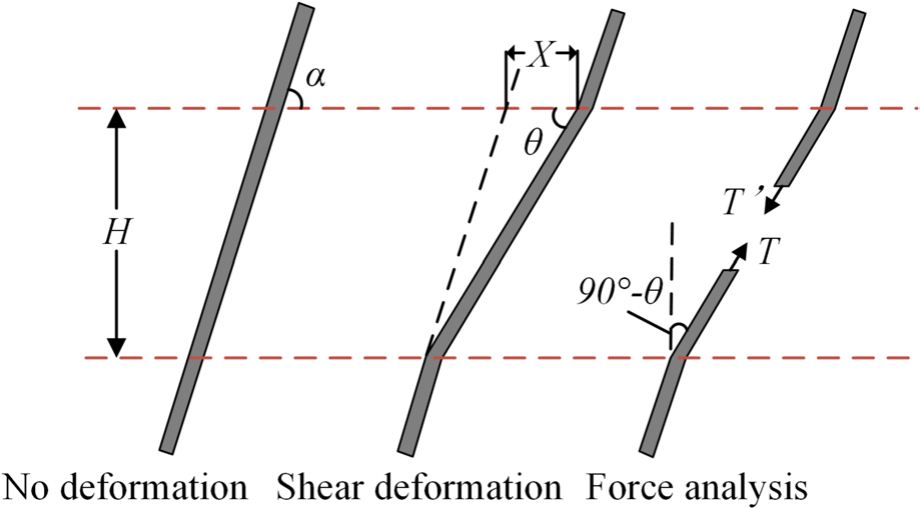
A mechanical model of root-soil interaction.

By establishing a mechanical interaction model between herbaceous plant roots and soil, the increase in shear strength ∆*τ*_*T*_ due to root reinforcement can be quantitatively described. Taking the analysis of a single root as an example, the simplified mechanical analysis diagram is shown in Fig. 1^23^. Assuming the thickness of the soil shear zone is *H*, the angle between the single root and the soil layer is *α*, and the shear deformation angle of the single root (shear strain) is *θ*, the additional shear strength ∆*τ*_*T*_ contributed by the single root to the soil can be expressed as:

2 $$\Delta \tau_{T} = \frac{T}{a}\sin \left( {90^{ \circ } - \theta } \right) + \frac{T}{a}\cos \left( {90^{ \circ } - \theta } \right)\tan \varphi$$

3 $$\theta = \arctan \left( {\frac{H}{{X + \frac{H}{\tan \alpha }}}} \right) = \arctan \left( {\frac{1}{{k + \left( {\tan \alpha } \right)^{ - 1} }}} \right)$$

In the equation: *T* represents the tensile force of a single root; *a* denotes the area of soil influenced by the single root; *X* is the horizontal displacement of the single root; and *k* is the shear deformation ratio, with the thickness of the soil shear zone *H*, where *k* = *X*/*H*.

If there are n roots acting within an area *A* of the soil, with the tensile forces of the roots represented by *T*_1_, *T*_2_, …, *T*_n_, the shear deformation angles by *θ*_1_, *θ*_2_, …, *θ*_n_, the horizontal displacements by *X*_1_, *X*_2_, …, *X*_n_, and the shear deformation ratios by *k*_1_, *k*_2_, …, *k*_n_, then the additional shear strength contributed by the root system to the soil can be expressed as:

4 $$\Delta \tau_{T} = \frac{{\sum {T_{i} \sin \left( {90^{ \circ } - \theta_{i} } \right)} }}{A} + \frac{{\sum {T_{i} \cos \left( {90^{ \circ } - \theta_{i} } \right)} }}{A}\tan \varphi$$

5 $$\theta_{i} = \arctan \left( {\frac{H}{{X_{i} + \frac{H}{{\tan \alpha_{i} }}}}} \right) = \arctan \left( {\frac{1}{{k_{i} + \left( {\tan \alpha_{i} } \right)^{ - 1} }}} \right)\;(i = {1},{2}, \ldots ,n)$$

By substituting Eq. (4) into Eq. (1), the general calculation formula for the shear strength *τ* of the root-soil composite can be obtained as:

6 $$\tau = \left( {\sigma \tan \left( {\varphi + \Delta \varphi_{m} } \right) + \frac{{\sum {T_{i} \cos \left( {90^{ \circ } - \theta_{i} } \right)} }}{A}\tan \varphi } \right) + \left( {c + \Delta c_{m} + \frac{{\sum {T_{i} \sin \left( {90^{ \circ } - \theta_{i} } \right)} }}{A}} \right)$$

From Eq. (6), it can be seen that the shear strength of the root-soil composite is mainly related to the internal friction angle, cohesion, and the tensile strength of the roots. In the vast majority of cases, during the process of pulling out the roots of herbaceous plants from the soil layer, the roots are pulled apart. Therefore, the tensile strength of the roots is less than the bonding and friction forces between the roots and the soil. The tensile strength of roots with different diameters can be measured experimentally in the laboratory.

If the proportion of roots in the root-soil composite is small, it can be assumed that the internal friction angle is still predominantly determined by the soil, and the increase in cohesion is mainly due to the mechanical effects of the roots on the soil. Consequently, Eq. (7) can be further simplified to:

7 $$\tau = \left( {\sigma + \frac{{\sum {T_{i} \cos \left( {90^{ \circ } - \theta_{i} } \right)} }}{A}} \right)\tan \varphi + \left( {c + \frac{{\sum {T_{i} \sin \left( {90^{ \circ } - \theta_{i} } \right)} }}{A}} \right)$$

### Root reinforcement mechanical model

For herbaceous plants with finer roots and shallow growth depths, the primary function is soil reinforcement, so a reinforced anti-sliding mechanical model is recommended; for those with thicker roots and deeper growth depths, the main function is anchorage, so an anchored anti-sliding mechanical model should be used.

(1) Reinforced anti-sliding mechanical model.

On a slope with an angle of *α*, assuming that the potential slip surface is parallel to the slope surface and the total weight of the potential sliding mass is *G*, the root-soil composite near the slip surface is relatively weak and its strength is similar to that of the native soil. Therefore, the mechanical properties of the root-soil composite can be neglected, and the mechanical effect of plant roots plays a key role in soil reinforcement and landslide resistance. Based on the above assumptions and in combination with Eq. (4), the residual sliding force *P*_*s*_ of the sliding mass can be expressed as:

8 $$P_{s} = G\sin \alpha - \left[ {G\cos \alpha \tan \varphi + cA_{s} + \sum {T_{i} \sin \left( {90^{ \circ } - \theta_{i} } \right)} { + }\sum {T_{i} \cos \left( {90^{ \circ } - \theta_{i} } \right)} \tan \varphi } \right]$$

Let $k = G\cos \alpha \tan \varphi + cA_{s}$; $R_{f} = \sin \left( {90^{ \circ } - \theta_{i} } \right) + \cos \left( {90^{ \circ } - \theta_{i} } \right)\tan \varphi$, generally, *R*_*f*_ can be taken as 1.0 (fully mobilized strength) or 1.2 (partially mobilized)^24^; $F = \sum {T_{i} }$, then the above equation can be simplified as:

9 $$P_{s} = G\sin \alpha - \left( {k + 1.2F} \right)$$

In the equation, *φ* denotes the internal friction angle of the potential sliding soil; *c* denotes the cohesion of the potential sliding soil; *A*_*s*_ denotes the area of the potential sliding surface; and $\sum {T_{i} }$ represents the total tensile force generated by the roots of herbaceous plants.

(2) Anchorage anti-sliding mechanical model.

**Fig. 2:**
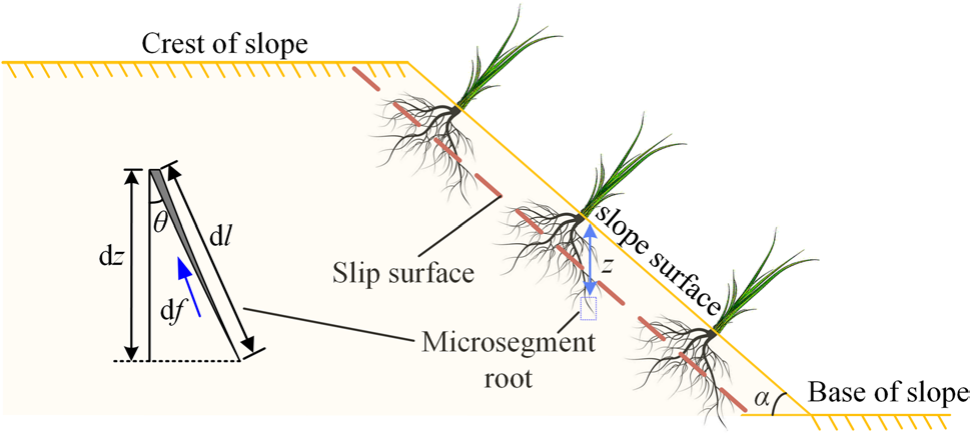
Schematic diagram of anti-slip of herbaceous plant roots.

On a slope with an angle of *α*, consider an arbitrary infinitesimal segment d*l* of a main or coarse root located at a depth *z* from the ground surface. Let *μ* denote the static friction coefficient between the root and the soil,* γ* the unit weight of the soil, and *θ* the inclination angle of the root segment, as shown in Fig. 2.

When the shallow soil of a slope undergoes critical sliding along the shear plane, the maximum static friction force d*f* experienced by a micro-segment root is:

10 $$df = \mu \gamma z \bullet 2\pi r \bullet dl$$

The vertical projection component of d*f* is:

11 $$df_{z} = df \bullet \cos \theta = \mu \gamma z \bullet 2\pi r \bullet dl \bullet \cos \theta = \mu \gamma z \bullet 2\pi r \bullet dz$$

If the average radius distribution function of the root system along depth *z* is *D*(*z*), and the root number distribution function along depth *z* is *N*(*z*), then the vertical component of the maximum static friction force *F*_*z*_ of the root system within the depth range *H* is given by:

12 $$F_{z} = 2\pi r\tan \left( {\varphi + \Delta \varphi_{m} } \right)\int_{0}^{H} {D\left( z \right)} \bullet N\left( z \right) \bullet z{\text{dz}}$$

In the formula, $\mu = \tan \left( {\varphi + \Delta \varphi_{m} } \right)$ represents the static friction coefficient, and $\varphi + \Delta \varphi_{m}$ denotes the internal friction angle of the root-soil composite. The component of the frictional resistance provided by the root system along the slope angle,* F*_*α*_, is given by:

13 $$F_{\alpha } = F_{z} \sin \alpha$$

If critical instability and sliding occur along the weak surface (sliding surface), and the residual thrust is parallel to the slope surface, the residual thrust *P*_*s*_ of the sliding mass can be calculated as:

14 $$P_{s} = G\sin \alpha - G\cos \alpha \tan \varphi - cA_{s}$$

To maintain the stability of the shallow slope soil, the following condition must be satisfied:

15 $$F_{\alpha } - P_{s} \ge 0\;or\;F_{\alpha } /P_{s} \ge 1$$

## Experimental study on the shear characteristics of root-soil complexes of herbaceous plants

### Sample collection

**Fig. 3:**
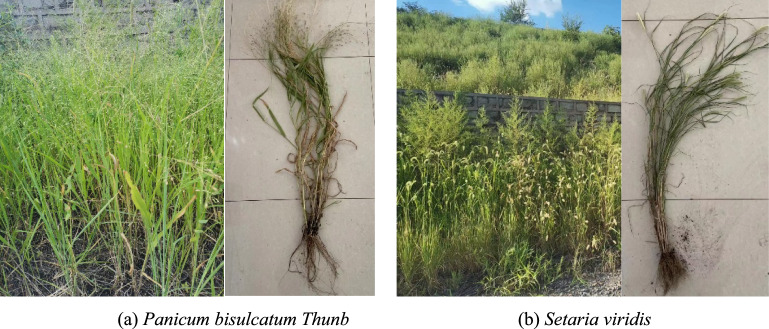
Object of study.

*Setaria viridis* is an annual late-spring herbaceous plant with flat leaves that have rough edges. It grows to a height of approximately 10–100 cm, with basal stems reaching 4 ~ 7 mm in diameter. This species is widely distributed in China, thriving in fields, roadsides, slopes, and various herbaceous plant communities. It is commonly found in loose, fertile soils rich in humus, such as sandy or clay soils, and exhibits strong environmental adaptability with low water requirements. Foxtail grass belongs to the Poaceae family and has a fibrous root system, primarily composed of fine roots. *Panicum bisulcatum Thunb*, also a member of the Poaceae family and classified under the Panicum genus, is an annual herbaceous plant. It has slender, relatively rigid culms that grow upright or with basal nodes that may root, reaching a height of 0.5–1 m. *Panicum bisulcatum Thunb* typically inhabits moist wastelands and primarily propagates through seeds. *Setaria viridis* and *Panicum bisulcatum Thunb* are the most common herbaceous species in the Haizhou mining area, exhibiting abundant growth and providing a sufficient supply of experimental materials, as shown in Fig. 3.

Both *Setaria viridis* and *Panicum bisulcatum Thunb*. are common herbaceous plants widely distributed in northeastern China, including the Haizhou mining area where our study was conducted. The plant materials were formally identified by the corresponding author Wenqi Wang based on morphological characteristics using standard taxonomic references. To ensure traceability, voucher specimens of both species have been deposited in the Herbarium of Liaoning Technical University (Fuxin, China). The accession numbers are: LNTU-FX-2024–004 for *Setaria viridis* and LNTU-FX-2024–005 for *Panicum bisulcatum Thunb*.

### Relationship between root content and depth

The study on the root growth patterns of herbaceous plants reveals that root diameter varies at different soil depths^25^. To characterize the root-soil composite properties at different depths, the root content is used as an indicator to determine the proportion of root-soil mixtures. Based on the actual root content in the shallow soil of the Haizhou open-pit mine slope, the root content ratio of the remolded soil is designed accordingly.

In this study, root content was defined as the dry mass of roots relative to the dry mass of the corresponding soil sample, expressed as a percentage (%). The calculation follows Eq. (16):

16 $$\eta { = }\frac{{m_{1} }}{{m_{2} }} \times 100\%$$

where $\eta$ is the root content (%), $m_{1}$ is the dry mass of roots (g), and $m_{2}$ is the dry mass of soil (g).

Field measurements were conducted using a profile method, with a 10 cm × 10 cm area sampled beginning 2 cm below the slope surface. For each plant, four horizontal profiles were excavated at 2 cm vertical intervals. Roots from each profile were carefully extracted, dried, and weighed. Soil dry density was measured and multiplied by layer volume to obtain soil dry mass. The natural root content at each depth was then calculated, and the mean values across five Setaria viridis plants were taken as the representative root content for that depth. The same procedure was applied to Panicum bisulcatum Thunb., and comparable root content profiles were obtained for this species.

**Fig. 4:**
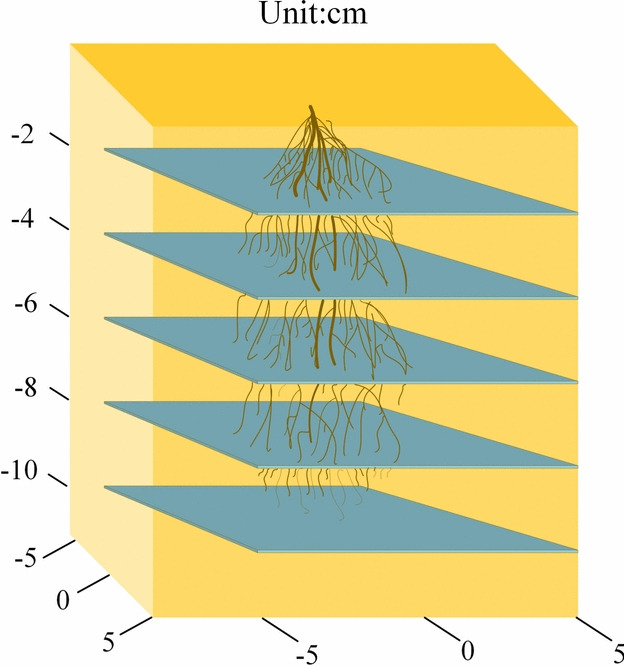
Schematic diagram of soil profile.

Field results showed that root content was significant only within the 0–8 cm depth range; below 8 cm, root content was negligible. Based on these statistics, the laboratory direct shear tests were designed with root contents of 0.4%, 0.3%, 0.2%, 0.1%, and 0%, corresponding to average root contents at depths of 0–2 cm, 2–4 cm, 4–6 cm, 6–8 cm. The soil profile is shown in Fig. 4, and the statistical results are presented in Table 1.

**Table 1:** Root system statistics.

Plant species	Soil depth(cm)	Average root content(%)
*Panicum bisulcatum Thunb*	0 ~ 2	0.381
	2 ~ 4	0.295
	4 ~ 6	0.204
	6 ~ 8	0.112
*Setaria viridis*	0 ~ 2	0.373
	2 ~ 4	0.289
	4 ~ 6	0.193
	6 ~ 8	0.097

### Specimen preparation and testing

The root content of the soil used in the experiment was designed based on the root content ratio of the undisturbed soil. Rooted and non-rooted soil samples were collected from the Haizhou open-pit coal mine. After being brought back to the laboratory, the roots were removed, the attached soil particles were washed off, and the roots were naturally air-dried until their weight stabilized. All soil samples were placed in an XMA-2000 electric hot-air drying oven and baked at 105 °C for 24 h to completely remove the moisture. After drying, the soil was sieved, and the roots were cut into 2 cm sections.

**Fig. 5:**
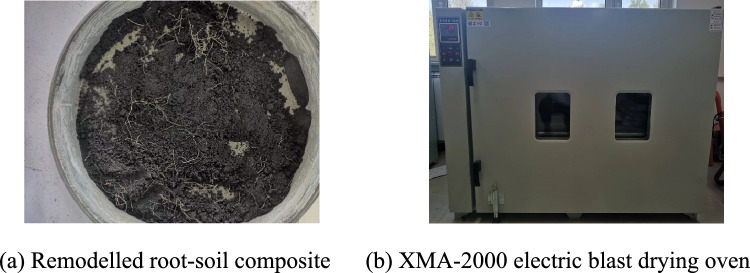
Shear specimen preparation.

Based on the measured moisture content, different water content conditions were established to simulate the root–soil reinforcement behavior under varying rainfall and weather scenarios^26^. Accordingly, three sets of remolded soil samples were prepared with water contents of 9%, 11%, and 13%. Shear samples with root content ratios of 0%, 0.1%, 0.2%, 0.3%, and 0.4% were prepared for each moisture condition, corresponding to root depths of 0–2 cm, 2–4 cm, 4–6 cm, and 6–8 cm, respectively. All tests were completed within three days to prevent root decay, ensuring the biological activity was maintained. The procedure is illustrated in Fig. 5.

The soil and roots were thoroughly mixed, and after compaction, four shear samples were prepared for each soil type using a ring cutter. During the test, different moisture content samples were prepared by adding water to the dried soil.

**Fig. 6:**
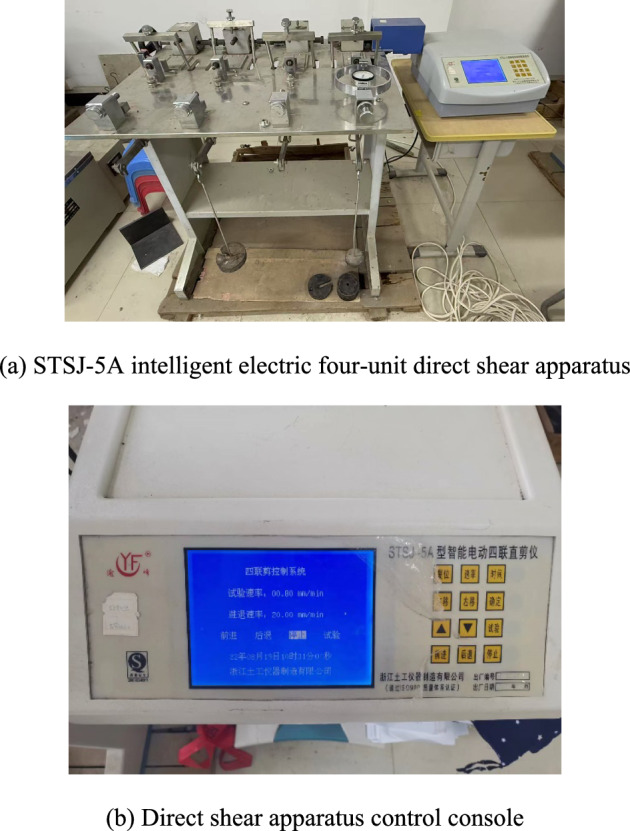
Test equipment of direct shear.

The shear strength of the root-soil composite was determined using a direct shear apparatus. The shear rate for the test specimens was set at 0.8 mm/min, with four samples tested in each group. Normal stress of 100, 200, 300, and 400 kPa, determined according to the testing standard and the apparatus loading specifications, were applied to the samples. During horizontal shearing, readings were recorded from the dial gauge, and the test was terminated when the values ceased to increase. The maximum value was recorded. The specific equipment is shown in Fig. 6.

### Shear failure characteristics of root-soil composite specimens

**Fig. 7:**
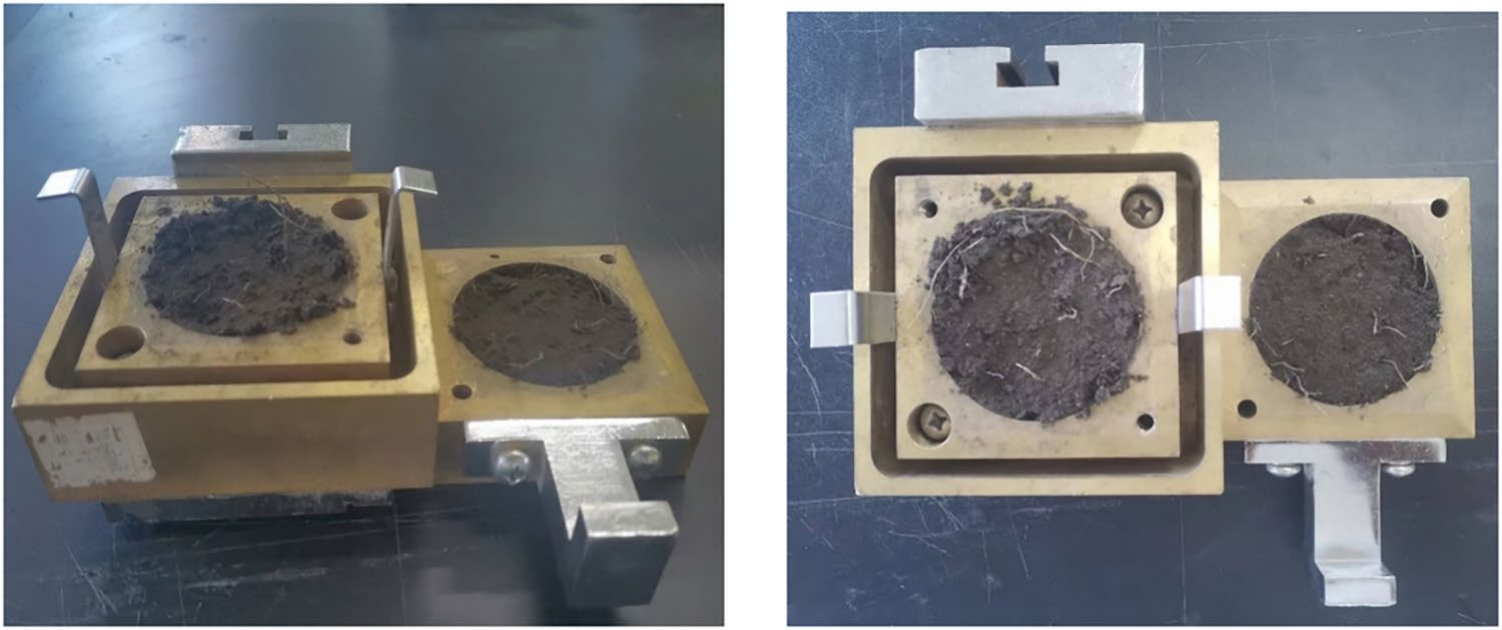
Shear failure of specimen.

As shown in Fig. 7, during the shear process of the specimens, most roots were pulled out, while root breakage was relatively rare. The primary reason for this is that the shear test of the remolded root-soil composite differs from in-situ shear tests. When the roots are extracted from the soil layer in the test area, the initial stress state between the roots and the soil is disrupted, leading to a higher degree of root vitality loss in the remolded root-soil composite. This reduces the ability of the roots to effectively reinforce the soil. Compared to the shallow root-soil composite in natural slopes, the interaction force between the remolded roots and soil is weaker. Consequently, under shear stress, roots in the remolded composite are more likely to be pulled out rather than sheared or broken. Therefore, in the indoor direct shear tests, the roots of Setaria viridis predominantly exhibited pullout behavior.

The reported cohesion intercepts are Mohr–Coulomb intercepts fitted over the selected normal stress range and may include apparent cohesion effects; the focus of this study is on the relative trends in shear strength enhancement due to roots rather than absolute cohesion values.

## Data processing and analysis

### Analysis of shear strength of root-soil composite under different moisture contents

**Fig. 8:**
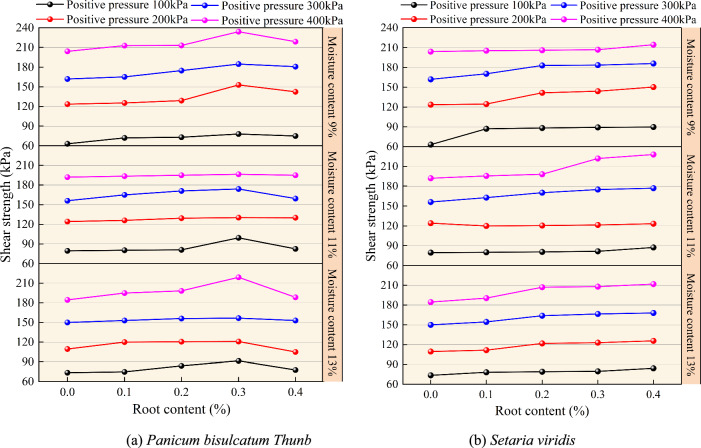
Shear strength of root-soil composite as a function of root content at different moisture contents.

The shear strength of the root-soil composite with different moisture contents for two herbaceous plants is shown in the curve in Fig. 8, as a function of root content. For *Setaria viridis* root-soil composite, the shear strength increased with the root content in the range of 0 ~ 0.4%. Under all four normal pressure conditions, the average increase in shear strength was around 10% for every 0.1% increase in root content. Specifically, under the condition of 11% moisture content and a normal pressure of 200 kPa, the shear strength at a root content of 0.2% increased by 0.5% compared to a root content of 0.1%, which was the smallest increase. At 9% moisture content and 100 kPa normal pressure, the shear strength at 0.1% root content increased by 38.1% compared to the pure soil, which was the largest increase. Similarly, under 9% moisture content and 100 kPa normal pressure, the shear strength of *Setaria viridis* root-soil composite increased by 42.3% compared to pure soil, with a corresponding root content of 0.4%.

For *Panicum bisulcatum Thunb*, there was an optimal root content of approximately 0.3%, and this optimal root content was not affected by changes in the moisture content of the root-soil composite. For *Setaria viridis*, the shear strength of the root-soil composite increased with root content in the range of 0 ~ 0.4%. Overall, the shear strength of the root-soil composite for both herbaceous plants decreased with increasing moisture content.

### Variation of shear strength parameters of root-soil composite at different soil depths

**Fig. 9:**
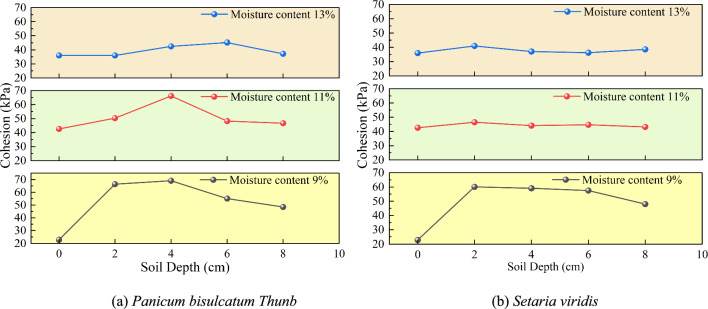
Relationship between soil depth and cohesion under different moisture contents.

**Fig. 10:**
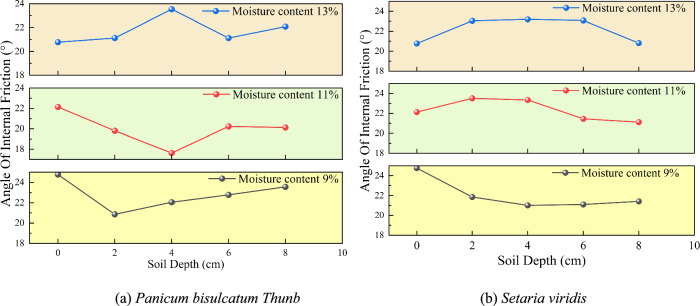
Relationship between soil depth and internal friction angle under different moisture contents.

Record the maximum shear stress of the specimens under different normal stresses to obtain the relationship curve between shear strength and vertical pressure for different samples. Analyze the shear strength parameters of the soil based on the Mohr–Coulomb strength criterion, as shown in Fig. 9 and Fig. 10.

The cohesion of the *Panicum bisulcatum Thunb* root-soil composite first increases and then decreases with depth, which may be related to the optimal root content in remolded soil. When the soil depth is 2 ~ 4 cm and the moisture content is 9%, the cohesion of the *Panicum bisulcatum Thunb* root-soil composite increases by 222.1% compared to rootless soil.

In contrast, the cohesion of the *Setaria viridis* root-soil composite decreases with depth. At a moisture content of 9% and a depth of 0 ~ 2 cm, the cohesion of the *Setaria viridis* root-soil composite reaches its maximum, increasing by 185.8% compared to rootless soil.

Compared to *Setaria viridis*, the internal friction angle of the *Panicum bisulcatum Thunb* root-soil composite shows a more significant variation with depth, indicating that the root system of *Panicum bisulcatum Thunb* has a greater influence on the internal friction angle of the soil than *Setaria viridis* roots.

## Analysis of herbaceous root-sliding resistance effect at different moisture contents

### Calculation method for slope stability factor

The slope stability factor, also known as the slope safety factor, is an important parameter for determining whether a slope will experience landslides or other forms of failure. The limit equilibrium method^27^ is a widely used approach for determining slope stability. This method uses the ratio of the resisting forces to the driving forces in the slope as the basis for judgment, i.e.:

17 $$K = \frac{{\int {(c + \sigma \tan \varphi )dA} }}{{\int {\tau dA} }}$$

When the slope stability factor is greater than 1.2, the slope is considered stable.

**Fig. 11:**
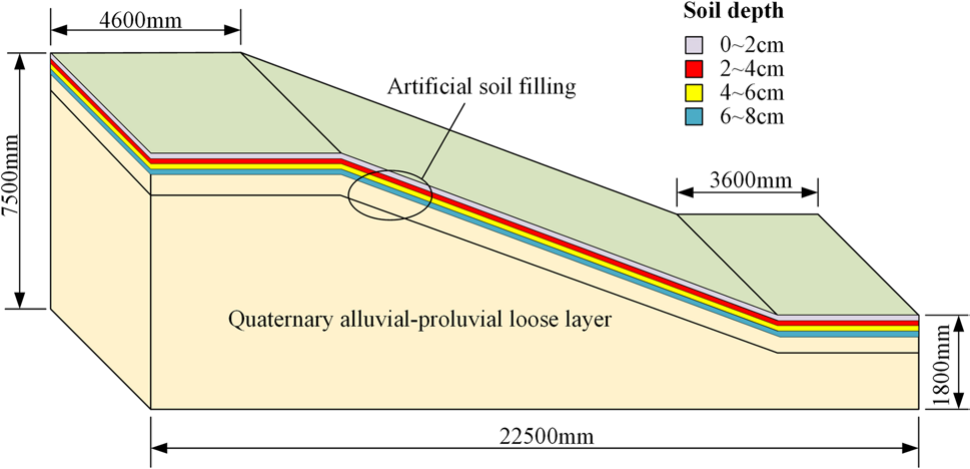
Geometric size of slope model.

In this study, GEO-SLOPE software was used, specifically the SLOPE/W module, to simulate the slope and calculate the slope stability factor using the limit equilibrium method. Based on the topography of the open-pit mine, a simplified slope model was established, as shown in Fig. 11. The slope soil layers are divided into artificial fill (Q_4_^ml^) and Quaternary alluvial and floodplain loose layers (Q_4_^apl^), with a surface fill thickness of 0.5 m.

As shown in Table 1, the root content in the soil changes with depth. Table 2 shows that the mechanical properties of the soil also vary with different root contents. To more accurately simulate the effect of roots on the soil, the surface soil within the root influence zone is divided into layers, with a thickness of 2 cm per layer, totaling 4 layers.

**Table 2:** Mechanical parameters of shear soil samples under different conditions.

Root types	Moisture content(%)	Root content(%)	Depth of root action(cm)	Angle of internal friction(°)	Cohesion(kPa)
Plain soil	9%	0	0	24.77	22.8
	11%	0	0	22.14	42.6
	13%	0	0	20.78	36.0
*Panicum bisulcatum Thunb*	9%	0.1%	6 ~ 8	23.56	48.5
		0.2%	4 ~ 6	22.78	55.1
		0.3%	2 ~ 4	22.05	69.1
		0.4%	0 ~ 2	20.86	66.4
	11%	0.1%	6 ~ 8	20.12	46.7
		0.2%	4 ~ 6	20.23	48.2
		0.3%	2 ~ 4	17.64	66.2
		0.4%	0 ~ 2	19.80	50.3
	13%	0.1%	6 ~ 8	22.09	37.2
		0.2%	4 ~ 6	21.13	45.2
		0.3%	2 ~ 4	23.54	42.5
		0.4%	0 ~ 2	21.13	36.0
*Setaria viridis*	9%	0.1%	6 ~ 8	21.42	48.0
		0.2%	4 ~ 6	21.11	57.5
		0.3%	2 ~ 4	21.02	59.1
		0.4%	0 ~ 2	21.86	60.1
	11%	0.1%	6 ~ 8	21.12	43.2
		0.2%	4 ~ 6	21.46	44.7
		0.3%	2 ~ 4	23.35	44.1
		0.4%	0 ~ 2	23.52	46.5
	13%	0.1%	6 ~ 8	20.82	38.6
		0.2%	4 ~ 6	23.09	36.3
		0.3%	2 ~ 4	23.20	37.1
		0.4%	0 ~ 2	23.06	41.0

The limit equilibrium method for calculating the slope stability factor requires three parameters: the weight of the soil, the cohesion, and the internal friction angle. Stokes^28^ found that the weight of the roots does not affect slope stability. Therefore, in this study, the effect of root weight is not considered in the calculation of slope stability.

The densities of the bedrock and surface soil, as well as the values of the cohesion and internal friction angle of the root-soil composite under different moisture contents and root contents (from Table 2), are assigned to the corresponding regions to simulate the effect of roots on the surface soil. The slip surface of the slope is determined, and using the software’s solving module, the slope stability factor under different root influences is obtained.

### Slope stability analysis

**Fig. 12:**
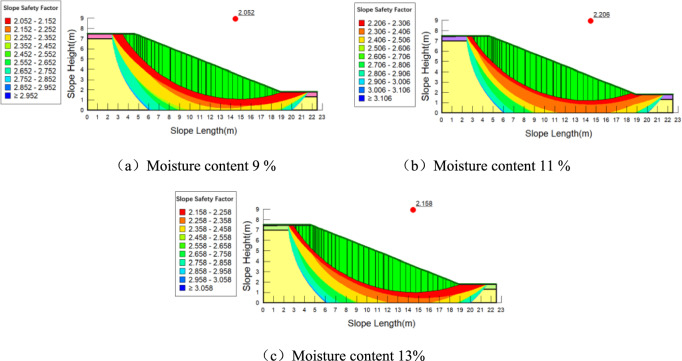
Stability analysis of plain soil slope under different moisture contents.

**Fig. 13:**
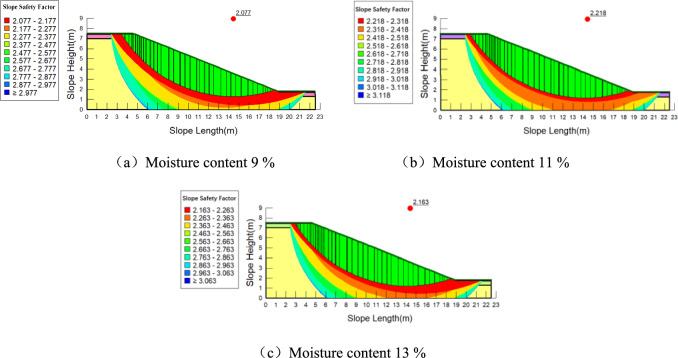
Stability analysis of slopes planted with *Panicum bisulcatum Thunb* under different moisture contents.

**Fig. 14:**
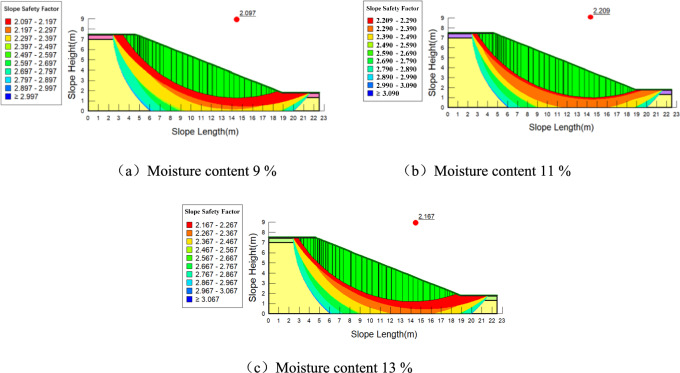
Stability analysis of slopes planted with Setaria viridis under different moisture contents.

The values of cohesion and internal friction angle at different depths from Table 2 are assigned to the model, and the range of the slope slip surface is determined. The calculation results are shown in Fig. 12, Fig. 13 and Fig. 14.

When the moisture content is between 9 and 13%, the slope stability factor first increases and then decreases. When the soil is particularly dry, an appropriate increase in moisture content can reduce the frictional resistance between soil particles, making them more easily movable and compacted, thereby increasing the dry density of the soil, enhancing shear strength, and improving slope stability. However, as the moisture content continues to increase, the cohesion between the soil particles decreases, leading to reduced shear strength and a decrease in the stability of the shallow soil layers of the slope. The stability trends for slopes with and without roots follow the same pattern with changes in moisture content.

Roots improve the stability of the slope at different moisture contents. The variation in slope stability factor with moisture content is shown in Table 3. However, it is important to note the following considerations: When the moisture content in the slope soil increases by the same amount, the increase in stability for slopes with different root types is generally smaller than that for bare soil slopes. This suggests that the influence of roots moderates the effect of moisture on slope stability, but this effect is limited.

**Table 3:** Rlationship between the stability coefficient of different types of slopes and the moisture content.

Type of slope	moisture content(%)	Slope stability factor
Plain soil slope	9%	2.052
	11%	2.206
	13%	2.158
Planting of *Panicum bisulcatum Thunb* slopes	9%	2.077
	11%	2.218
	13%	2.163
Planting of *Setaria* slopes	9%	2.097
	11%	2.209
	13%	2.167

Global factor of safety and reinforcement depth: The factor of safety reflects the balance of forces or moments along the critical slip surface, which in this model is located several meters below the ground surface. The modeled root system is restricted to a depth of approximately 0.08–0.10 m. Given this, root reinforcement is unlikely to influence the global factor of safety for deep‐seated slip surfaces. Shallow roots may contribute to the stability of surficial soil layers or reduce erosion but cannot significantly alter the mechanics of deeper slip surfaces.

magnitude of differences: differences in factor of safety between vegetated and bare slopes are relatively small. Such differences may arise from modelling uncertainties, moisture variation, rounding errors, or numerical setup, rather than reflecting a significant mechanical effect of roots.

Implications for root reinforcement: While shallow roots may help reduce surface erosion or shallow slips, their influence on deep‐seated stability is negligible. Therefore, the slight increase in factor of safety for vegetated slopes should be interpreted with caution. These results do not provide strong evidence of root reinforcement for deep slip surfaces within the limits of the current modelling setup.

In summary, moisture content has a clear influence on slope stability, with an optimal range where stability is maximized. Vegetation appears to buffer moisture-related stability changes in the surface soil to some extent, but the modeled shallow root layer does not significantly affect the global factor of safety for deep‐seated slopes. Consideration of this limitation is essential for the accurate interpretation of the results.

### Limitations

The present study was conducted on remolded root–soil composite under controlled laboratory conditions, with root geometry altered by cutting and drying. Matric suction was not measured or controlled, and the reinforcement depth was shallow (≤ 10 cm), which may not fully capture field-scale slope processes. The applied normal stresses in the shear tests were higher than typical near-surface overburden in the rooted zone; therefore, the absolute cohesion values should not be directly generalized to shallow field conditions. The numerical modelling is intended as an illustrative application and can serve as a clear example of the limitations of such approaches; extrapolation to natural slopes should be made cautiously given the site-specific soil conditions and the limited number of plant species tested.

## Discussion

Previous studies have shown that the root content in the soil is a significant factor affecting the soil’s shear strength. This paper finds that the shear strength of the root-soil composite increases as the root content in the soil increases until it reaches the optimal root content. This is consistent with Wang Yuanzhan’s findings^29^, where it was observed that for remolded grass root-reinforced soil, there is an optimal root content that maximizes its strength. Similarly, Liao Bo^30^ and others found that there is an optimal root content that results in the maximum shear strength of the root-soil composite.

In this study, remolded soils were used for indoor direct shear testing based on several considerations. Firstly, laboratory conditions offer better control over variables such as temperature, humidity, and vegetation interference, thereby improving the repeatability and comparability of experimental results. Secondly, field conditions are often highly variable, and soil moisture is significantly influenced by rainfall and evaporation, making it difficult to achieve precise, multi-gradient water content control. In contrast, the use of remolded soil in laboratory settings allows moisture content to be adjusted as needed to meet specific test design requirements. Additionally, due to the complex topography of mining slopes, construction disturbances, and high safety risks, conducting in situ shear tests presents substantial technical challenges and operational difficulties. And the mechanical responses of remolded soils to changes in root content and moisture conditions are largely consistent with those of undisturbed soils. Therefore, remolded soil can serve as an effective medium for studying the shear performance of root–soil composites^31,32^.

In real-world slope conditions, prolonged or intense rainfall can significantly reduce soil strength and may even trigger slope failures. Some herbaceous species—particularly those with sparse or highly permeable root systems—may facilitate water infiltration and accelerate slope saturation, thereby exerting a negative influence on slope stability^33^. For instance, Wang^34^ demonstrated through simulated experiments in expansive soils that high-intensity rainfall weakens the stabilizing effect of plant roots in the shallow layer, underscoring the need for future studies to incorporate rainfall–infiltration processes into comprehensive stability assessments. Future experimental and numerical studies should therefore introduce rainfall infiltration models to more accurately evaluate the risk of such "bio-facilitated infiltration" mechanisms.

Many shallow landslides and surficial instabilities occur within the upper 0.5–1.0 m of slopes, particularly in fill slopes or erosion-prone areas^35–37^. Herbaceous plant roots can enhance shallow-layer shear strength and reduce surface runoff, thereby delaying the onset and progression of slope failure. Zhou^38^ further noted that even when roots do not penetrate potential slip surfaces, their homogenized reinforcement in the upper soil layers can effectively retard the development of shallow failures. This is especially relevant for practical applications such as mine reclamation and vegetation-based slope stabilization.

It should be emphasized that this study specifically focused on two common herbaceous species (*Panicum bisulcatum Thunb* and *Setaria viridis*) that dominate the slopes of the Fuxin Haizhou open-pit coal mine. Woody plants, by contrast, typically possess both finer roots and thicker anchoring roots, thereby exerting dual reinforcement and anchorage effects. Although this was beyond the scope of the present experiments, such species merit further investigation in future studies.

Therefore, although this study is based on controlled laboratory tests, the findings hold valuable implications for the selection, spatial arrangement, and surface reinforcement design of herbaceous vegetation in engineering practice. Given the limited rooting depth of herbaceous species, future research by our group will explore the reinforcement potential of woody plants or mixed-root systems to achieve combined shallow and deep soil stabilization. On the slopes of the Fuxin Haizhou open-pit mine, the commonly occurring herbaceous vegetation consists mainly of two species (*Panicum bisulcatum Thunb* and *Setaria viridis*), while woody plants are scarce and were not included in this study. Although woody plants can provide both reinforcement and anchoring effects due to their fine and thick roots, this study focused on the existing herbaceous vegetation. Future work may consider incorporating woody species to further investigate ecological engineering effects.The present study is particularly applicable to surface slope protection in mining areas, shallow topsoil layers, or early-stage ecological restoration projects.

## Conclusion

This study analyzes the soil stabilization effect of the root systems of two herbaceous plants at different moisture contents and root contents through remolded root-soil composite indoor direct shear tests. The slope protection effect is analyzed using numerical simulation methods, and the following conclusions are drawn:

Plant roots primarily provide additional cohesion to the soil through reinforcement, limiting lateral deformation of the soil, thereby increasing the shear strength of the soil. Before the root content in the soil reaches the optimal level, the shear strength of the root-soil composite increases with the increase in root content.

The shear strength of the remolded root-soil composite of *Panicum bisulcatum Thunb* reaches its maximum at a root content of 0.3%, which can be considered the optimal root content for *Panicum bisulcatum Thunb*. Under the same moisture content conditions, the shear strength of the *Panicum bisulcatum Thunb* root-soil composite increases by approximately 24.9% compared to the rootless soil. The soil stabilization ability of *Setaria viridis* is greatly affected by moisture content. At a moisture content of 9%, the shear strength of the composite is maximized, increasing by 41.7% compared to the rootless soil.

When the slope moisture content is between 9 and 13%, the slope stability factor of the *Setaria viridis* slope, *Panicum bisulcatum Thunb* slope, and bare soil slope first increases and then decreases. Under the same moisture content conditions, the slope stability factor of the root-containing soil slopes is higher than that of the bare soil slope, indicating that within this moisture range, the presence of *Panicum bisulcatum Thunb* roots increased peak shear strength by up to 24.9%, and *Setaria viridis* roots by up to 41.7%, relative to rootless soil, thereby improving slope stability.

## Data Availability

The datasets used and/or analysed during the current study are available from the corresponding author upon reasonable request.

## References

[1] Xiao, D. et al. Key Climate Factors in Species Introduction for Ecological Reconstruction of the Dump Site of Shenhua Baorixile Open-pit Mining. *J. South China Normal Univ. Nat. Sci. Ed.* **53**, 10. 10.6054/j.jscnun.2021045 (2021).

[2] Ma, P., Xia, D., Xu, W., Cheng, H. & Cai, C. Numerical simulation of the influence of plant root reinforcement on gully erosion stability. *J. Disaster Prev. Mitig. Eng.* **39**, 354–364 (2019).

[3] Wu, M., Zhou, C., Wang, L. & Tan, C. Numerical simulation of the influence of roots and fissures on hydraulic and mechanical characteristics of soil. *Rock Soil Mech.* **40**, 519–526. 10.16285/j.rsm.2018.1728 (2019).

[4] Kong, G., Wen, L., Liu, H. & Wang, C. Strength properties of root compound soil and morphological observation of plant root. *Rock Soil Mech.* **40**, 3717–3723. 10.16285/j.rsm.2018.1338 (2019).

[5] Cheng, L., Yao, L., Zhang, S., Hao, Y. & Song, Q. Experimental study on the root morphology impact on soil strength. *J. Nat. Disasters* **27**, 40–49. 10.13577/j.jnd.2018.0106 (2018).

[6] Ouyang, M. Study on the influence of root system configuration regulation on slope stabilization performance. MSc thesis, Central South Univ Forestry Technol, (2021).

[7] Li, Z. et al. Enhancing plant root-soil reinforcement capability on slopes through root configuration regulation. *Rock Soil Mech.* **42**, 3271–3280. 10.16285/j.rsm.2021.0660 (2021).

[8] Ji, X. & Yin, P. Influence of different root growth stages on the slope anti-erosion performance. *J. Cent. South Univ. Forestry Technol* **40**, 144–150. 10.14067/j.cnki.1673-923x.2020.12.017 (2020).

[9] Xu, H. et al. Influence of root morphology and hierarchical of roots on the mechanical characteristics of root-soil composites. *Chin. J. Geotech. Eng.* **44**, 926–935. 10.11779/CJGE202205016 (2022).

[10] Shen, Z. et al. Mechanical characteristics of root systems and shear strength of root-soil composites of four plant species in the Yellow River source region. *Soil Water Conserv. China* **7**, 49–52. 10.14123/j.cnki.swcc.2021.0168 (2021).

[11] Li, Y., Chen, J., Chen, X. & Wang, T. Study on soil reinforcement effects of five slope-protecting herbaceous plant roots. *Soil Water Conserv. China* **1**, 41–45. 10.14123/j.cnki.swcc.2021.0014 (2021).

[12] Wang, X. et al. Effect of root architecture on rainfall threshold for slope stability: Variabilities in saturated hydraulic conductivity and strength of root-soil composite. *Landslides* **17**, 1965–1977. 10.1007/s10346-020-01422-6 (2020).

[13] Sun, Q., Ge, Y., Chen, P., Liang, X. & Du, Y. Evaluation of factors influencing the shear strength of root-soil composites of typical plants in the Wenchuan area. *J. Soil Water Conserv.* **36**, 58–65. 10.13870/j.cnki.stbcxb.2022.01.009 (2022).

[14] Mao, X., Xu, P., Cao, Y., Fan, M. & Yang, J. Experimental study on shear strength of plant roots in open-pit coal mine wastelands. *Sci. Soil Water Conserv. China* **17**, 103–110. 10.16843/j.sswc.2019.06.013 (2019).

[15] Zhu, J., Wang, Y., Wang, Y. & Ma, C. Analyses on Root reinforcement Mechanism Based on plant Growth Process and Optimization of Wu Model. *Sci. Silv. Sin.* **54**, 49–57 (2018) (**CNKI:SUN:LYKE.0.2018-04-006**).

[16] Lv, J., Wang, W., Dai, T. & Liu, G. Experimental Study on the Effect of Soil Reinforcement and Slip Resistance on Shallow Slopes by Herbaceous Plant Root System. *Sustainability* **16**, 3475. 10.3390/su16083475 (2024).

[17] Li, X. et al. Numerical Study on Soil Reinforcement Mechanism Combined with Artemisia in Loess slope Protection. *J. Gansu Sci.* **34**, 95–99. 10.16468/j.cnki.issn1004-0366.2022.01.015 (2022).

[18] Ekanayake, J. & Phillips, C. A method for stability analysis of vegetated hillslopes: an energy approach. *Can. Geotech. J.* **36**, 1172–1184. 10.1139/t99060 (1999).

[19] Ekanayake, J. & Phillips, C. Slope stability thresholds for vegetated hillslopes: a composite model. *Can. Geotech. J.* **39**, 849–862. 10.1139/t02-026 (2002).

[20] Pollen, N. & Simon, A. Estimating the mechanical effects of riparian vegetation on stream bank stability using a fiber bundle model. *J. Water Resour. Res.* **41**(7), 226–244. 10.1029/2004WR003801 (2005).

[21] Greenwood, J. R. SLIP4EX – A Program for Routine Slope Stability Analysis to Include the Effects of Vegetation, Reinforcement and Hydrological Changes. *Geotech. Geol. Eng.* **24**, 449–465. 10.1007/s10706-005-4156-5 (2006).

[22] El Hariri, A. E. E. & Kiss, P. Review on soil shear strength with loam sand soil results using direct shear test. *J. Terramech.* **107**, 47–59. 10.1016/j.jterra.2023.03.003 (2023).

[23] Zhou, D. & Zhang, J. *Vegetation Slope Protection Engineering Technology* (People’s Transportation Publishing House, 2002).

[24] Duncan, J. M. & Chang, C. Y. Nonlinear Analysis of Stress and Strain in Soils. *J. Soil Mech. Found. Div.* **96**, 1629–1653. 10.1061/JSFEAQ.0001458 (1970).

[25] Yang, Q., Zhang, C., Yao, S. & Jiang, J. Root Distribution and Root Cohesion of Two Herbaceous Plants in the Loess Plateau of China. *Sustainability* **14**, 8053. 10.3390/su14138053 (2022).

[26] Fan, C. C. & Su, C. F. Effect of soil moisture content on the deformation behaviour of root-reinforced soils subjected to Shear. *Plant Soil* **324**, 57–69. 10.1007/s11104-008-9856-1 (2009).

[27] Kumar, S., Choudhary, S. S. & Burman, A. Three-Dimensional Slope Failure Response Based on Limit Equilibrium Method. *Transp. Infrastruct. Geotech.* **11**, 2854–2884. 10.1007/s40515-024-00386-7 (2024).

[28] Stokes, A. et al. How Vegetation Reinforces Soil on Slopes; Springer: Dordrecht. *Netherlands*10.1007/978-1-4020-6676-4_4 (2008).

[29] Wang, Y. et al. Experimental research on influence of root content on strength of undisturbed and remolded grassroots-reinforced soil. *Chinese J. Geotech. Eng.* **37**, 1405–1410. 10.11779/CJGE201508007 (2015).

[30] Liao, B., Liu, J. & Zhou, H. Effects of the Infiuence of Root Content on the Shear Strength of Root-Soil Composite of Bischofia javanica. *J. Soil Water Conserv.* **35**, 104–110. 10.13870/j.cnki.stbcxb.2021.03.015 (2021).

[31] Jia, F. & Xu, H. On comparative test of dynamic tests of undisturbed soil and remolded Soils. *Shanxi Arch.* **43**, 65–66. 10.13719/j.cnki.cn14-1279/tu.2017.11.037 (2017).

[32] Lai, J. et al. Experimental Study on Shear Strength of Saturated Remolded Loess. *PLoS ONE* **17**(7), e0271266. 10.1371/journal.pone.0271266 (2022). 35834541 10.1371/journal.pone.0271266 PMC9282495

[33] Gong, C. et al. Herbaceous Vegetation in Slope Stabilization: A Comparative Review of Mechanisms, Advantages, and Practical Applications. *Sustainability* **16**, 7620. 10.3390/su16177620 (2024).

[34] Wang, Y., Xu, W., Wang, Z. & Zhu, Y. The Impact of Vegetation Roots on Shallow Stability of Expansive Soil Slope under Rainfall Conditions. *Appl. Sci.* **13**, 11619. 10.3390/app132111619 (2023).

[35] Schwarz, M., Lehmann, P. & Or, D. Quantifying lateral root reinforcement in steep slopes-from a bundle of roots to tree stands. *Earth. Surface Processes&Landforms.* **35**, 354–367. 10.1002/esp.1927 (2010).

[36] Kim, K.-S., Jeong, S.-W., Song, Y.-S., Kim, M. & Park, J.-Y. Four-Year Monitoring Study of Shallow Landslide Hazards Based on Hydrological Measurements in a Weathered Granite Soil Slope in South Korea. *Water* **13**, 2330. 10.3390/w13172330 (2021).

[37] Milledge, D. G., Bellugi, D., McKean, J. A., Densmore, A. L. & Dietrich, W. E. A multidimensional stability model for predicting shallow landslide size and shape across landscapes. *J. Geophys. Res. Earth Surf.* **119**(11), 2481–2504. 10.1002/2014JF003135 (2014). 26213663 10.1002/2014JF003135 PMC4508911

[38] Zhou, X. et al. The Shear Strength of Root-Soil Composites in Different Growth Periods and Their Effects on Slope Stability. *Appl. Sci.*10.3390/app131911116 (2023).

